# Impact of Biometric Patient Data, Probiotic Supplementation, and Selected Gut Microorganisms on Calprotectin, Zonulin, and sIgA Concentrations in the Stool of Adults Aged 18–74 Years

**DOI:** 10.3390/biom12121781

**Published:** 2022-11-29

**Authors:** Magdalena Jendraszak, Mirosława Gałęcka, Małgorzata Kotwicka, Andreas Schwiertz, Aleksandra Regdos, Michalina Pazgrat-Patan, Mirosław Andrusiewicz

**Affiliations:** 1 Chair and Department of Cell Biology, Poznan University of Medical Sciences, Rokietnicka 5D, 60-806 Poznań, Poland; 2 Institute of Microecology, Sielska 6, 60-129 Poznań, Poland; 3 Institute of Microecology, Auf den Lüppen 8, 35745 Herborn, Germany

**Keywords:** calprotectin, zonulin, secretory immunoglobulin A (sIgA), mucosal barrier, gut permeability, stool biomarker

## Abstract

Alterations to the intestinal barrier may be involved in the pathogenesis of various chronic diseases. The diagnosis of mucosal barrier disruption has become a new therapeutic target for disease prevention. The aim of this study was to determine whether various patient demographic and biometric data, often not included in diagnostic analyses, may affect calprotectin, zonulin, and sIgA biomarker values. Stool markers’ levels in 160 samples were measured colorimetrically. The analysis of twenty key bacteria (15 genera and 5 species) was carried out on the basis of diagnostic tests, including cultures and molecular tests. The concentrations of selected markers were within reference ranges for most patients. The sIgA level was significantly lower in participants declaring probiotics supplementation (*p* = 0.0464). We did not observe differences in gastrointestinal discomfort in participants. We found significant differences in the sIgA level between the 29–55 years and >55 years age-related intervals groups (*p* = 0.0191), together with a significant decreasing trend (*p* = 0.0337) in age-dependent sIgA concentration. We observed complex interdependencies and relationships between their microbiota and the analyzed biomarkers. For correct clinical application, standardized values of calprotectin and sIgA should be determined, especially in elderly patients. We observed a correlation between the composition of the gut community and biomarker levels, although it requires further in-depth analysis.

## 1. Introduction

Each individual is inhabited by a unique and complex gut ecosystem, combining different microorganisms that form a multifaceted microenvironmental community and impact the physiology of health and disease [1]. In recent years, the diversity of the gut microbiome and its impact on the health of the host have been the subjects of concentrated research efforts. The intestinal bacterial community is involved in a variety of essential processes in the human body, including the delivery and functions of enzymes that are not encoded by the human genome, the breakdown and absorption of nutrients, the production of vitamins and hormones, and the development of resistance to pathogen colonization. An increasing number of reports demonstrate that a well-balanced community of gut microorganisms may play a crucial role in promoting host health and well-being. At the same time, disturbances in the gastrointestinal (GI) microbiota can result in a wide variety of diseases [2,3,4]. However, the vast number of commensal microorganisms living in the GI tract pose a risk of infection. To this end, the primary function of intestinal epithelial cells is to form a barrier that maintains symbiotic homeostasis between gut bacteria and the human body. Current data highlight that the intestinal barrier is involved in positive and negative interactions with microbes. It allows immune cells to adapt to intestinal commensal bacteria, enabling the development of immune and food antigen tolerance, thus ensuring the proper functioning of the body [5,6,7,8].

The intestinal mucosal barrier is a complex, multi-layered system of physical, immunological, and microbial components that play a fundamental role in separating the inside of the body from the outside environment. This effect is enhanced by the secreted mucus and the production of secretory immunoglobulin A (sIgA), antimicrobial peptides, and proteins. The functional intestinal barrier acts as a selective filter that facilitates the absorption of nutrients, electrolytes, and water from the intestinal lumen into the host’s circulation. It tightly regulates the passage of proinflammatory molecules, potentially harmful microorganisms, toxins, and antigens through the intestinal epithelium into the body. This paracellular permeability is controlled by multiprotein complexes called tight junctions, located near the apical surfaces of adjacent epithelial cells [7,9,10]. Disturbances in the gut barrier caused by poor nutrition, infection, or other diseases can lead to increased epithelial permeability, also known as “leaky gut,” in which molecules can pass without restriction across the epithelium. The integrity and permeability of the intestinal barrier are critical for the organs’ homeostasis, while damage or dysfunction has local and systemic consequences. These are mainly associated with the direct contact of bacteria, bacterial metabolites, or their components with epithelial cells and, consequently, their translocation from the intestinal environment into the systemic circulation. Direct contact with bacteria/bacterial metabolites leads to the activation of immune cells and results in the secretion of proinflammatory mediators that extend the inflammatory process in the bowel or more distant organs. However, the dynamic relationship between the microbiota and the host environment, age, diet, and health status complicate the identification of specific bacterial species closely related to immune regulation [7,9,11,12,13].

Previous investigations have shown that altered intestinal microbiota can influence the development of local inflammation of the intestines and may coincide with chronic systemic inflammation [14,15]. Currently, there is speculation that an energy-rich, high-fat diet or excessive gluten consumption may adversely affect gut microbiota composition [16,17,18,19,20]. This leads to long-term exposure to high concentrations of bacterial lipopolysaccharides (LPS), resulting in the disruption of integrity and permeability of the intestinal barrier. Part of the outer membrane of the cell wall of Gram-negative bacteria, LPS has been identified as a potent mediator that can initiate an inflammatory process via toll-like receptors (4). It also appears that dietary fat can modulate intestinal permeability. It achieves this by overexpressing barrier-disrupting mediators such as interleukins (especially IL-1β, IL-6, IL-17, and IL-18), tumor necrosis factor-alpha, or interferon-gamma and by reducing the level of cytokines (e.g., IL-2 or IL-10) that support mucosal barrier homeostasis [5,21,22,23]. In addition, experimental data have shown that the overgrowth of pathogenic Gram-negative bacteria and the presence of LPS in the intestinal environment may reduce the expression and phosphorylation of tight-junction proteins, namely occludin and zonula occludens-1, and trigger the release of zonulin from the mucosal barrier, increasing the leakage of particles through the epithelium [16,19,22,23,24,25,26].

Maintaining intestinal barrier homeostasis is crucial for protecting the body from inflammation or infection. In the last decade, it was believed that increased intestinal permeability may be involved in the multifactorial pathogenesis of various chronic diseases, including obesity, type 2 diabetes, inflammatory bowel disease (IBD), celiac disease, autism, Alzheimer’s disease, and Parkinson’s disease [5,13,23,27]. Therefore, the methodological tools for investigating intestinal barrier function are developing rapidly, and the clinical utility of diagnostic tests that examine intestinal permeability have become a new therapeutic target for disease prevention and treatment.

In recent years, several molecules have been identified as potential prognostic and diagnostic biomarkers for epithelial cell integrity, enteritis, and intestinal immunity to provide rapid, non-invasive, and cheap methods. Currently, the most promising markers of intestinal inflammation are calprotectin (CAL), lactoferrin, and elastase. However, other molecules have been proposed; e.g., sIgA, defensins, and cathelicidins have all been highlighted as markers of intestinal immunity. Additionally, as intestinal permeability markers, zonulin (ZON), claudin-3, alpha-1-antitrypsin, or fatty acid-binding proteins (FABPs) have also been indicated [10,13,28,29]. However, it should be noted that most of these still require laboratory testing. Of note, large-scale, in-depth research is needed to identify clinically relevant bioindicators of gut barrier function that might have prognostic or diagnostic value.

This study was performed due to insufficient and divergent data on the concentration of markers of intestinal barrier integrity, permeability, and immunity such as zonulin, calprotectin, and sIgA in adults’ stool. We aimed to determine whether the levels of biomarkers can correlate with other additional factors, often not included in the diagnostic analysis, such as sex, age, and BMI. In addition, we wanted to establish whether there is an association between selected gut microbes, probiotic intake, GI dysfunction, and biomarker concentrations

## 2. Materials and Methods

We would like to emphasize that the results presented by us were not based on a clinical trial. All statistical analyses were performed based on self-report questionnaires and diagnostic results obtained at the Institute of Microecology (Poznan, Poland). The Institute of Microecology is a laboratory that performs commercial diagnostic tests in the field of gut microbiota assessment. Participants whose results were included in our analysis reported for commercial tests assessing the levels of biomarkers and the state of the gut microbiota. Written informed consent was obtained from participating patients.

### 2.1. Participants

This study aimed to compare 160 stool samples from women and men, screened for markers of intestinal function (calprotectin, sIgA, and zonulin) and selected gut microorganisms at the Institute of Microecology (Poznan) between 27 February 2020 and 18 April 2021. Before the diagnostic testing, the participants completed a self-reporting questionnaire regarding their age, sex, weight, height, overall health, acute and chronic diseases, probiotic or/and antibiotic supplementation, radiotherapy/chemotherapy treatment, and whether they were pregnant or breastfeeding. Additionally, questions concerned the occurrence of GI symptoms such as abdominal pain, constipation, diarrhea, and flatulence; functional disorders, e.g., irritable bowel syndrome (IBS) and small intestinal bacterial overgrowth (SIBO); and organic diseases, namely coeliac disease, inflammatory bowel diseases, lactose intolerance, and pancreas or liver diseases. The information included in the questionnaires was verified directly, by e-mail, or by telephone interview. Participants declared that all disorders were confirmed by standard or genetic diagnostic tests and imaging tests. A clinical evaluation established IBS in accordance with a modified Rome criteria [30]. A breath test diagnosed SIBO after lactulose ingestion according to hydrogen production. However, to form a homogeneous group, patients with organic diseases were excluded from further analysis. In addition, exclusion criteria included cancer history, autoimmune diseases, Lyme disease, diabetes mellitus, use of any antibiotic and drugs within three months before testing, general poor health, pregnancy, and breastfeeding. BMI was defined as body weight divided by the square of body height and was expressed in units of kg/m^2^ based on the weight in kilograms and height in meters.

Some participants declared that they had taken various probiotics (Vivomixx, Sanprobi, Dicoflor, Enterol). The number of strains in the probiotics ranged from one (*Saccharomyces boulardii* or *Lactobacillus plantarum* 299v) to nine bacteria strains. The multi-strain probiotics contained mainly *Lactobacillus* spp. and *Bifidobacterium* spp. and ranged from 2.5 to 10 × 10^9^ colony-forming units (CFU)/per gram. The duration of probiotic supplementation was heterogeneous. However, to create a homogeneous group, we limited it to those who had been taking probiotics for one month or more, as according to some reports, this time could impact the functioning of the intestines and the condition of the microbiota [31,32,33].

**Figure 1:**
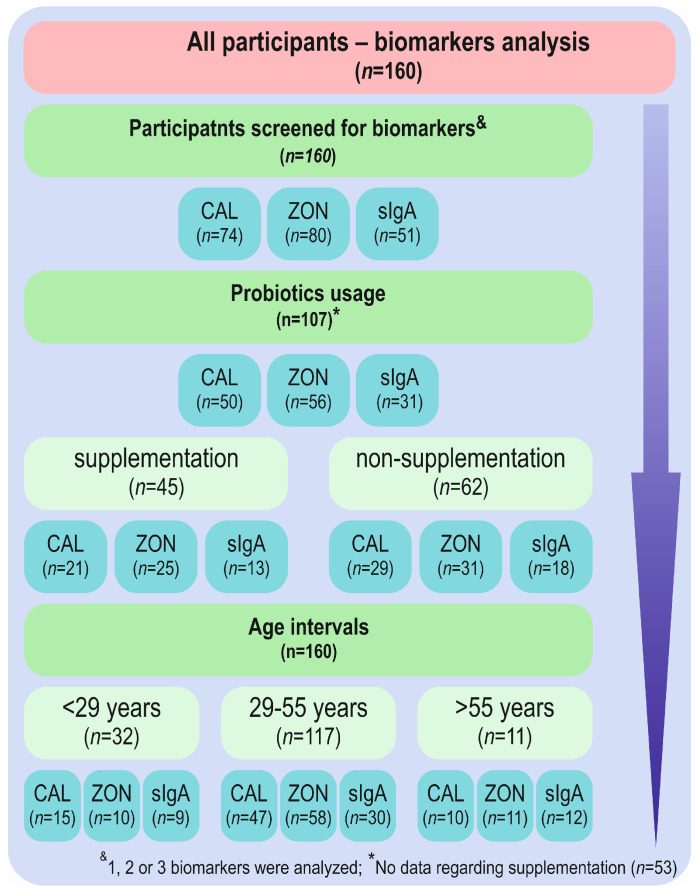
Study flow chart. CAL—calprotectin; ZON—zonulin; sIgA—secretory immunoglobulin A; *n*—number of studied cases.

Based on the self-report questionnaire, groups were created for further statistical analysis. We segregated groups according to probiotic supplementation (non-supplementation (n = 62), supplementation (n = 45)) and accordingly distinguished by Youden’s *J* index age intervals: <29 years (n = 32), 29–55 years (n = 117), and >55 years (n = 11) groups (Figure 1) and GI inconveniences (reported problems (n = 90) and no issues reported (n = 60)). Additionally, the GI disorders reported by participants, which included IBS (n = 21), SIBO (n = 9), diarrhea (n = 37), constipation (n = 47), and flatulence (n = 75), were taken into account (Supplementary Materials).

The study was conducted according to the criteria set out in the Helsinki Declaration. Before participating in the study, each subject was given and signed informed consent, and identifying information was removed from each sample. The protocol was approved by the Sciences Bioethics Committee of the Poznan University of Medical Sciences for the ethical treatment of people participating in biomedical research. However, it was concluded that this is not a clinical trial and does not have the characteristics of a medical experiment (date of approval: 8 December 2020). In accordance with local IRB regulations, our research requires fully anonymized participant data. All diagnostic tests were performed under the supervision of a laboratory diagnostics expert at the Institute of Microecology in Poznań.

### 2.2. Materials and Procedures

#### 2.2.1. Collection and Preparation of Stool Samples

Stool samples were collected at home in a sterile fecal tube by the participants and delivered to the Institute of Microecology (Poznan) within 24 h by a qualified door-to-door shipping service.

After visual inspection of the samples, biomarkers were analyzed with ready-to-use kits by following the manufacturer’s recommendations. Additionally, all samples underwent examination of selected bacteria. The tests were based on microbiological cultures and quantitative polymerase chain reactions (qPCR) according to the KyberKompact Pro Protocol developed by the Institute of Microecology in Herborn, Germany. The analysis of enteric microbes was based on a diagnostic microbiome test that allows detection and identification of foundational and keystone bacteria (15 genera and 5 species) in the intestinal ecosystem. Microbial analysis was performed on selective and differential/chromogenic agar plates and included: *Escherichia coli*, *Escherichia coli* (non-lactose fermenting—NLF), *Pseudomonas* spp., *Klebsiella* spp., *Enterobacter* spp., *Citrobacter* spp., *Enterococcus* spp., *Bifidobacterium* spp., *Bacteroides* spp., *Lactobacillus* spp., H_2_O_2_ *Lactobacillus, Clostridium* spp., *Clostridium difficile, Faecalibacterium prausnitzii*, *Akkermansia muciniphila*, *Proteus* spp*., Providencia* spp*., Morganella* spp*., Serratia* spp*.,* and *Hafnia alvei.* In addition to bacterial subsets, fecal fungi *Candida* spp. and molds were also determined. In addition, the total bacteria count (TBC) was estimated. The *Proteus* spp., *Providencia* spp., *Morganella* spp., *Serratia* spp., *Hafnia alvei*, and molds were excluded from the statistical analysis due to the lack of detection.

#### 2.2.2. Biomarkers Analysis

##### Calprotectin

ScheBo Master Quick-Prep (cat. no.: B-CAL-SO50-U; BÜHLMANN Laboratories AG, Schönenbuch, Switzerland), a multi-functional stool sample extraction system, was used for stool samples preparation. Fecal calprotectin quantification was performed according to the Quantum Blue^®^ fCAL extended in vitro diagnostic test manufacturers’ protocol (cat. no.: LF-CALE25-U; BÜHLMANN Laboratories AG, Schönenbuch, Switzerland). The selective CAL antigen level was measured in 60 μL of fecal sample diluted in extraction buffer by sandwich immunoassays. The test membrane was coated with a monoclonal capture antibody (mAb), highly specific for calprotectin. The conjugate release pad deposited the gold-colloids-conjugated monoclonal detection secondary antibody. The calprotectin/anti-calprotectin gold conjugate bound to the anti-calprotectin antibody on the test membrane (test line), while the remaining free anti-calprotectin gold conjugate bound to the goat anti-mouse antibody coated on the control line. Quantum Blue^®^ Reader (order code: BI-POCTR-ABS; BÜHLMANN Laboratories AG, Schönenbuch, Switzerland) quantifies the intensity of the test and control lines. The reference values for analyzed biomarkers are shown in Table 1.

##### Zonulin

Zonulin concentration was examined using the IDK^®^ Zonulin ELISA kit (cat. no. K 5600; Immundiagnostik AG, Bensheim, Germany) according to the manufacturer’s protocol using 15 mg of patient stool sample. Firstly, a biotinylated zonulin tracer was added to samples, standards, and controls. Then, aliquots of the treated samples, standards, and controls were incubated (1 h, room temperature, 350 rpm, horizontal shaker) in the wells of microtiter plates coated with anti-zonulin polyclonal antibodies. Samples were then washed five times in 250 μL wash buffer, and the remaining liquid was absorbed. Peroxidase-labeled streptavidin (100 µL), which binds to the biotinylated zonulin tracer, was then added to each well. The microplate was incubated, washed, and drained as described previously. Samples were incubated with 100 μL substrate solution for 15 min, and then the reaction was terminated with an acidic stop solution. The absorption was determined immediately at 450 nm against a reference wave of 620 nm using Infinite^®^ F50 compact ELISA absorbance microplate reader (TECAN, Männedorf, Switzerland). According to the manufacturer’s guidelines and the results presented by others [34,35], the median concentration of 61 ng/mL (±46 ng/mL) is estimated to be the health normal range for zonulin. The reference values are shown in Table 1.

##### Secretory Immunoglobulin A

The sIgA ELISA Kit (cat. no.: IC6100; ImmuChrom GmbH, Heppenheim, Germany) was used to quantitatively determine sIgA in stool and was carried out according to the manufacturer’s protocol. Fecal samples (100 mg) were mixed with 5 mL wash buffer, and 1 mL was transferred into an Eppendorf vial and centrifuged (10 min 2000× *g*, room temperature). The supernatant was diluted 1:250 in wash buffer, and 100 μL of the sample was processed using the sIgA ELISA assay to determine the human IgA secretion according to the sandwich principle. In short, after the washing step, a peroxidase-labeled detection antibody was added. The second washing step was followed by incubation with substrate solution. All samples were analyzed using an Infinite^®^ F50 compact ELISA absorbance microplate reader (TECAN, Männedorf, Switzerland) at 450 nm. The reference values are shown in Table 1.

#### 2.2.3. Microbiological Identification of Selected Microorganisms

Twenty-five milligrams of sample were diluted 1:10 in 0.85% sterile NaCl solution, suspended by vortexing, and subsequently plated on selective and differential agar medium plates. Then, the samples were inoculated on the following media: Columbia blood agar (TBC; Becton Dickinson, Heidelberg, Germany), Chromid CPS agar (*Escherichia coli*, *Proteus*, *Enterococcus, Klebsiella*, *Enterobacter*, *Serratia,* and *Citrobacter;* BioMerieux, Durham, NC, USA), Rogosa TMB HPR agar (*Lactobacillus*; Heipha, Eppelheim, Germany), *Bifidobacterium* agar (*Bifidobacteria*; Becton Dickinson, Heidelberg, Germany), Schaedler agar (*Bacteroides*; Heipha, Eppelheim, Germany), and SPM agar (*Clostridium* spp.; Heipha, Eppelheim, Germany). Plates were incubated under either aerobic or anaerobic conditions at 37 °C for 24 and 48 h. Both cultures and microscopic observations determined the presence of fecal fungi. Fecal samples were then suspended in 0.85% sterile NaCl solution containing trypsin, which is responsible for the breakdown of food residues in feces and the release of fungal cells and antibiotics (penicillin-streptomycin), which inhibit the growth of bacteria, and inoculated into two Sabouraud agar plates with further antibiotics (gentamicin and chloramphenicol). After 2–5 days, yeast colonies were transferred to differential plates that were assigned to the taxonomy species group. Molds were examined by morphological observation following 5–7 days of incubation [36].

QPCR was used to analyze anaerobic, unculturable bacteria such as *Akkermansia muciniphila* and *Faecalibacterium prausnitzii* and to determine *Clostridium difficile* numbers.

All counts were expressed as the numbers of log_10_ CFU per 1 g of sample. The reference values are presented in Table 2. The assessment of the selected intestinal microorganisms and the determination of the “normal” value were performed by the Institute of Microbiology in Herborn, Germany. Values and qualifications of the groups of protective, immunostimulating, and potentially pathogenic microorganisms were determined on the basis of population samples and available literature reports.

#### 2.2.4. DNA Isolation and Quantitative PCR Analysis

Bacterial DNA from stool samples was extracted using RIDA^®^ Xtract kit following the manufacturer’s instructions (cat. no.:PGZ001; R-Biopharm AG, Darmstadt, Germany). For bacteria quantity estimation, qPCR was performed with the use of *RIDA^®^GENE Akkermansia muciniphila*, *RIDA^®^GENE Faecalibacterium prausnitzii,* and *RIDA^®^GENE Clostridium difficile* kits according to the manufacturer’s protocol (cat. nos.: PG0145, PG0155, and PG0835, respectively; R-Biopharm AG, Darmstadt, Germany). The total reaction volume was 25 μL containing 19.9 μL reaction mix, 0.1 μL Taq Polymerase, and 5 μL DNA extract. Samples were treated as follows: initial denaturation (1 min, 95° C), 45 cycles of denaturation (15 s, 95 °C), and annealing/extension (30 s, 60 °C). The standard curve was generated with DNA standard A (5 × 10^2^ copies/reaction), DNA standard B (5 × 10^4^ copies/reaction), and DNA standard C (5 × 10^6^ copies/reaction). The fluorescence measurements were performed in the RotorGene thermal cycler (QIAGEN, Manheim, Germany). The final number of bacteria/gram of stool was obtained by multiplying by 200 due to the dilution factor of the stool sample during extraction. The reference values are presented in Table 2.

### 2.3. Statistical Analyses

Statistical analyses were performed using Statistica software v.13.3.704.0 (TIBCO Software, Tulsa, OK, USA) and PQStat software v.1.8.0.476 (PQStat Software, Poznan, Poland). The graphical visualization of the data was performed with JMP Pro software v.16.2.0 (570548) (SAS Institute, Cary, NC, USA) R software v.4.2.0 [37], RStudio version 2022.02.1 + 461 (Free Software Foundation, Boston, MA, USA) [38], ggplot2 package [39,40], and GGally package v2.1.2 [41].

The distribution of continuous variables was assessed with a Shapiro–Wilk test. As the data were not normally distributed, a nonparametric, 2-sided Kruskal–Wallis test with Dunn’s post hoc test for multiple comparisons and Mann–Whitney U test (tied-ranks-adjusted) for two groups were used. Jonckheere–Terpstra trend test was estimated for the ordinal grouping variables. The nonparametric Spearman’s rank correlation test was applied to determine the strength of a link between microbe species, and missing data were pairwise deleted. For multiple hypothesis testing, sequential modified Bonferroni correction was applied. Youden’s *J* statistic was used to estimate the age index (referring to probiotics usage presence). For individual comparisons, a *p*-value < 0.05 was considered significant. The logarithmic values of the microorganisms and biomarker levels were used for the graphical presentation of the data. The results are described using descriptive statistics and shown as mean ± standard deviation (M ± SD) or median [lower–upper quartile] (Me [Q1–Q3]).

## 3. Results

### 3.1. Participants’ Characteristic

Our study included 49 male and 111 female participants that did not differ in age (M ± SD = 38 ± 11.7 vs. 41 ± 13.1; *p* > 0.05). The male and female participants did differ in body mass index (*p* = 0.0413; Me = 23.89, Q1–Q3: [21.22–26.86] vs. 21.94 [19.02–24.80]). When groups were considered for their probiotics supplementation, the groups did not differ significantly in age and BMI (*p* > 0.05; Supplementary Table S1).

### 3.2. Differences in Biomarker and Microbiota Level in Analyzed Stool Samples

**Figure 2:**
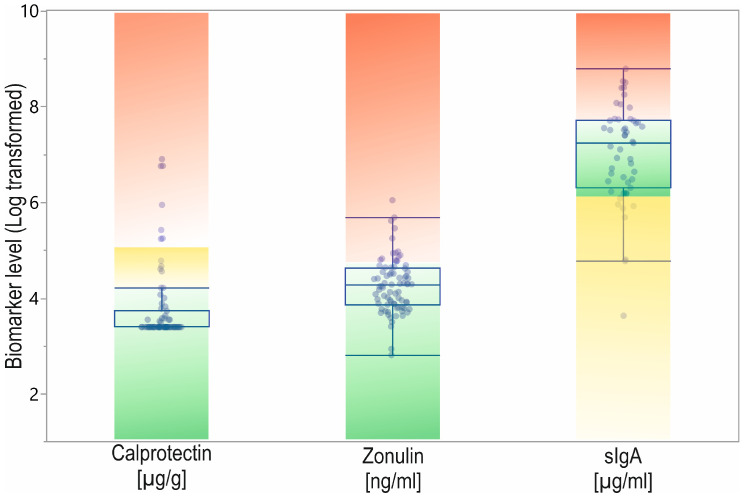
The calprotectin, zonulin, and sIgA data from patients with reference range. Designation: green—normal level; yellow—subnormal or gray-zone; red—elevated level.

All biomarker concentrations were within normal limits in most participants (Table 1, Figure 2). In the case of calprotectin, 81% of patients were in the normal range, and only 19% had borderline or elevated levels. Zonulin was in the normal reference range for 79% of participants and elevated in the remaining 21%. Considering sIgA, 24% of patients showed lower than normal levels, 43% were in the normal range, and 34% had elevated levels.

**Figure 3:**
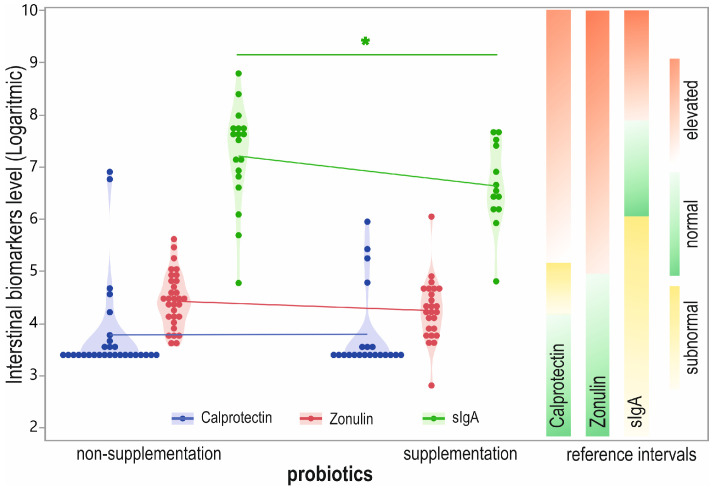
Stool calprotectin, zonulin, and sIgA levels (log transformed) in participants distinguished by probiotic supplementation with biomarkers reference range. Designation: green—normal level; yellow—subnormal or gray-zone; red—elevated level; * *p* < 0.05.

The results were similar when comparing the normal and abnormal levels of calprotectin in the non-probiotic-supplemented and supplemented patients (normal: 83% vs. 81%) and similar concerning zonulin (normal: 71% vs. 80%). Results for sIgA in the non-probiotics-supplemented group demonstrated subnormal levels for 17% of participants, normal levels in 44%, and elevated levels in 39% of patients. The percentages in the probiotics-supplemented group were 31%, 54%, and 15%, respectively (Figure 3).

We also found a significant difference in sIgA level between the two groups (*p* = 0.0464), with lower levels in the probiotic-supplementing group (Figure 3). The levels of remaining biomarkers did not differ between the groups (Supplementary Table S2), and we did not observe differences in participants suffering gastrointestinal discomfort. A significant difference was observed in the *E. coli* NLF level (*p =* 0.0291) between the probiotic-supplemented and non-supplemented groups (Supplementary Table S3), with the higher result being from the non-probiotic-supplemented group.

**Figure 4:**
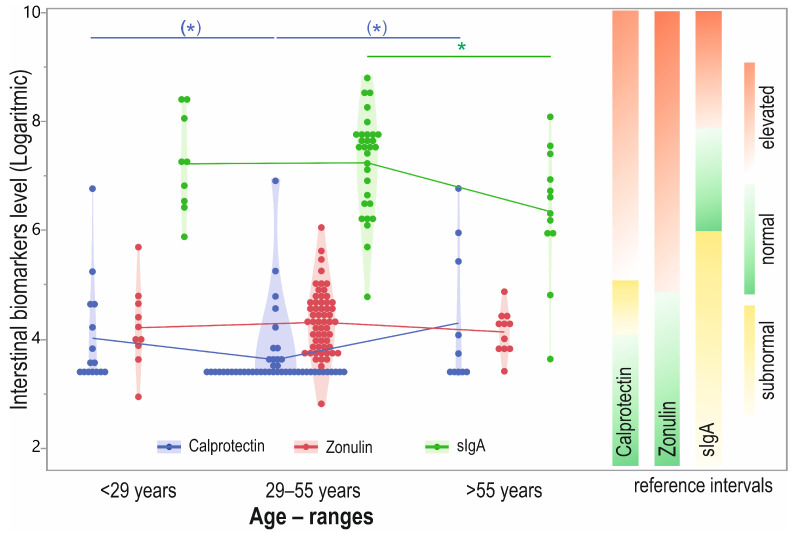
Stool calprotectin, zonulin, and sIgA level (log transformed) in participants distinguished by age intervals with biomarkers reference range. Designation: green—normal level; yellow—subnormal or gray-zone; red—elevated level; * *p* < 0.05; (*) *p* = 0.1–0.05.

We observed significant differences between the groups when considering biomarker concentrations in different age intervals (distinguished by Youden’s *J* index) (*p* = 0.047), with the difference observed between the 29–55 years and >55 years groups (*p* = 0.0191). Furthermore, the Jonckheere–Terpstra test showed a significant trend (*p* = 0.0337) of an age-dependent decrease in sIgA levels. Additionally, the level of calprotectin was trending toward differential expression between age groups, but this did not reach significance (*p* = 0.0557). The post hoc test demonstrated that the 29–55 years interval showed the lowest median level compared to <29 years (*p* = 0.0518) and >55 years (*p* = 0.0748), but again, the difference was not significant (Figure 4).

**Figure 5:**
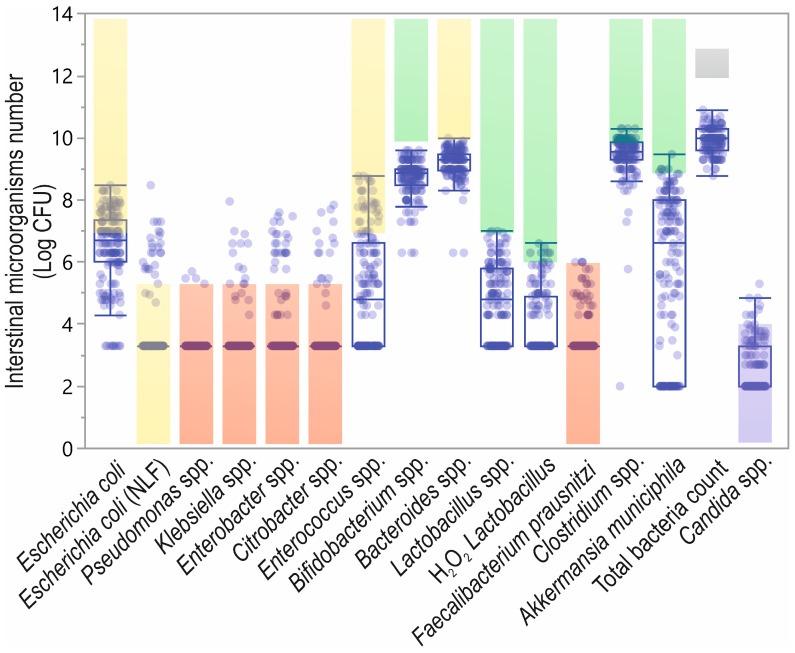
Microbiota species in analyzed patients with reference values. The shadow boxes represent the reference values (according to the Institute of Microecology (Herborn, Germany): green—protective species; yellow—immunostimulating species; red—potentially pathogenic species; grey—total bacteria count; violet—*candida* spp.); Log CFU—logarithmic transformed colony-forming units.

In most patients, the pathogenic microbiota level was in the normal range. Conversely, considering the immunostimulating microbiota species, the concentration was only in the normal range for *E. coli* NLF, in half of the patients for *E. coli* and both *Enterococcus* spp. and *Bacteroides* spp. were below the normal range. In the case of protective species, only half of the patients were in the normal range for *Faecalibacterium prausnitzii,* and lower than normal values were found in the cases of other studied species (Table 2, Figure 5).

### 3.3. Relationships between Biomarkers and Microorganisms

**Figure 6:**
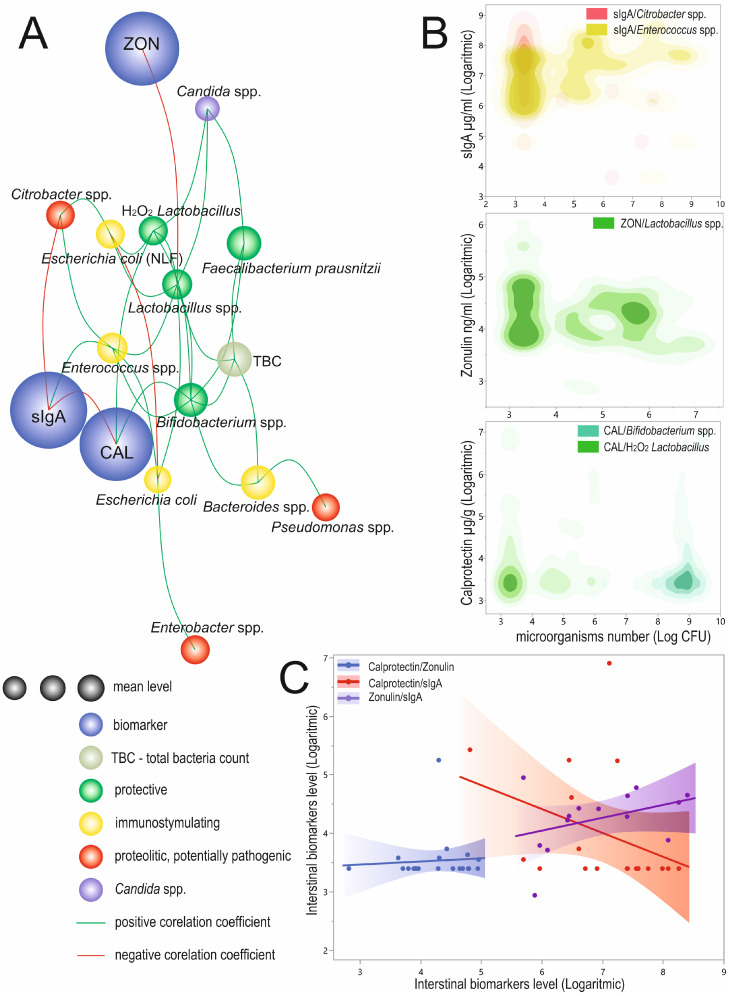
Linkage distances between analyzed biomarkers and analyzed microorganisms. The length of each line represents the strength of the correlation coefficient (**A**). In the case of the circle diameter of microorganisms, the circle size represents the mean level. The biomarker circle size was not scaled. Significant correlations of analyzed biomarkers and microorganism species (**B**) and correlation between logarithmic transformed biomarker levels (**C**). CAL—calprotectin; sIgA—secretory immunoglobulin A; ZON—zonulin; TBC—total bacteria count; NLF—non-lactose fermenting; Log CFU—logarithmic transformed colony-forming units.

We observed twenty-eight significant correlations between biomarkers and/or selected microorganisms (Figure 6A, Supplementary Table S4). We observed a significant, positive, and weak correlation between the calprotectin and H_2_O_2_ *Lactobacillus* (R = 0.25; *p* = 0.0343) and *Bifidobacterium* spp. (R = 0.25; *p* = 0.003). The sIgA was significantly, positively, and weakly correlated with *Enterococcus* spp. (R = 0.28; *p* = 0.0447) and negatively, moderately correlated with *Citrobacter* spp. (R = −0.39; *p* = 0.0042). The significant correlation between zonulin and *Lactobacillus* spp. was weak and negative (R = −0.27; *p* = 0.0145; Figure 6B, Supplementary Table S4). Both calprotectin and sIgA showed a significant, mutual, strong, and negative correlation (R = −0.6; *p* = 0.0085; Figure 6C, Supplementary Table S4).

Apart from biomarkers, we observed twenty-two significant correlations between microorganisms of different strengths (Figure 6A, Supplementary Table S4). Interestingly, the *Escherichia coli* level was very weakly correlated with NLF *E. coli* (R = −0.17; *p* = 0.0319). The strongest significant and positive correlations were observed between *Lactobacillus* spp. and H_2_O_2_ *Lactobacillus* (R = 0.61; *p* < 0.0001) with both *Bacteroides* spp. and *Bifidobacterium* spp. and TBC (R = 0.58; *p* < 0.0001 and R = 0.50; *p* < 0.0001, respectively). The remaining seventeen significant correlations were weak or very weak (Figure 6A, Supplementary Table S4).

#### Correlation of Biomarkers with Probiotics Supplementation

**Figure 7:**
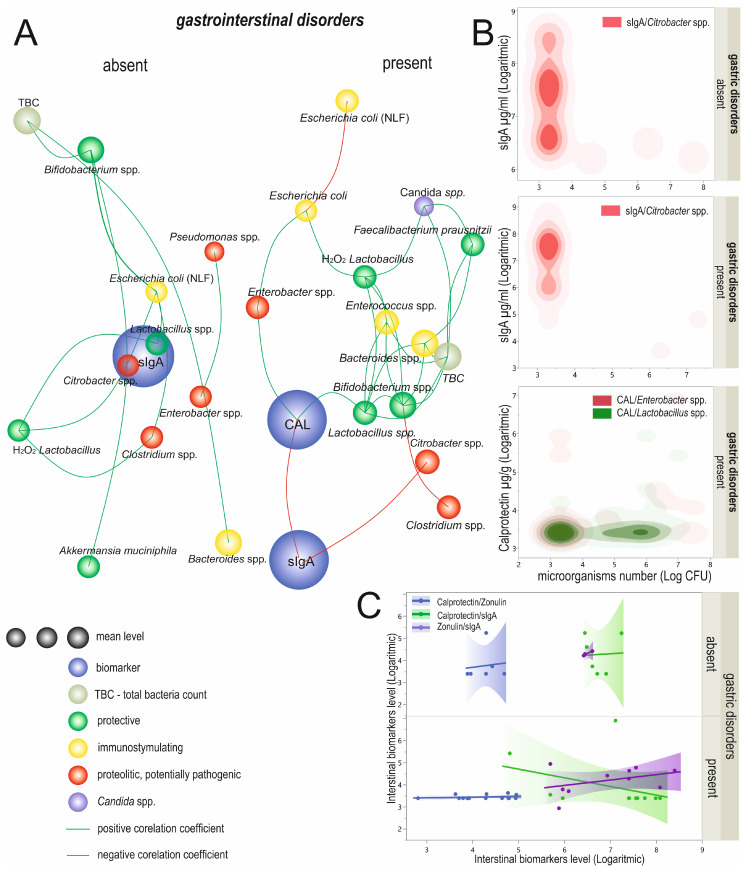
Linkage distances between analyzed biomarkers and selected microorganisms in groups distinguished according to probiotics usage (**A**). The length of each line represents the strength of the correlation coefficient. In the case of the circle diameter of microorganisms, the circle size represents the mean level. The biomarker circle size was not scaled. Significant correlations of analyzed biomarkers and microorganism species (**B**) and correlation between logarithmic transformed biomarkers’ levels (**C**). CAL—calprotectin; sIgA—secretory immunoglobulin A; ZON—zonulin; TBC—total bacteria count; NLF—non-lactose fermenting; Log CFU—logarithmic transformed colony-forming units.

When considering probiotics usage, we observed twelve significant correlations between biomarkers and/or analyzed microorganisms in the group who did not report probiotics supplementation and sixteen in those that reported probiotics supplementation (Figure 7A, Supplementary Table S5). We observed a significant, positive, and moderate correlation between the calprotectin and *Klebsiella* spp. in participants declaring probiotic supplementation (R = 0.44; *p* = 0.0203). Additionally, zonulin levels correlated negatively and moderately with *Lactobacillus* spp. (R = −0.43; *p* = 0.0164) and positively and weakly with *Clostridium* spp. (R = 0.36; *p* = 0.0468; Figure 7B; Supplementary Table S5). In the group of patients who supplemented their diet with probiotics, calprotectin proportionally correlated with three analyzed microorganisms, moderately with *E. coli* and *Lactobacillus* spp. (R = 0.43; *p* = 0.0496 and R = 0.47; *p* = 0.0304, respectively) and strongly with H_2_O_2_ *Lactobacillus* spp. (R = 0.64; *p* = 0.0024). Furthermore, we observed a moderate and positive zonulin and *E. coli* (NLF) correlation (R = 0.40; *p* = 0.0468; Figure 7B; Supplementary Table S5). We did not observe significant correlations between biomarker levels in the non-probiotics-taking or the probiotics-supplementation group (*p* > 0.05; Figure 7C).

We observed nine significant positive correlations between the microorganisms in the non-probiotics-supplementing group of participants. Strong correlations characterized *Bacterioides* spp and TBC (R = 0.67; *p* < 0.0001) as well as H_2_O_2_ *Lactobacillus* and Lactobacillus spp. (R = −0.62; *p* < 0.0001). Moderate dependencies were observed in the cases of *Bacteroides* spp. and *Bifidobacterium* spp. (R = 0.51; *p* < 0.0001) and *Bifidobacterium* spp. and TBC (R = 0.51; *p* < 0.0001). The other correlations in this group were weak (Figure 7C; Supplementary Table S5). On the other hand, thirteen microorganism-to-microorganism correlations were significant in patients supplementing their diet with probiotics. Of these, the majority of the correlations were positive; only *E. coli* correlated negatively with *E. coli* (NLF), with a moderate and negative result (R = −0.40; *p* = 0.0068). Strong correlations were observed of *Bacteroides* spp. with TBC and H_2_O_2_ *Lactobacillus* with *Lactobacillus* spp. (in both cases R = 0.62 *p* < 0.0001). Positive and moderate correlations characterized *Bifidobacterium* spp. with TBC (R = 0.52; *p* = 0.0003), *Candida* spp. with *Lactobacillus* spp. (R = 0.41; *p =* 0.0052) and H_2_O_2_ *Lactobacillus* (R = 0.45; *p* = 0.0027), and *Faecalibacterium prausnitzii* with TBC (R = 0.43; *p* = 0.0031). Six other significant correlations were positive and weak (Figure 7A; Supplementary Table S5).

## 4. Discussion

In the past decade, the significance of the mucosal barrier to the pathogenesis of disease has attracted the attention of not only researchers but also the wider community. There is growing evidence that a malfunction of the intestinal mucosa is a causative factor in the pathogenesis of various intestinal and parenteral diseases such as IBD, IBS, celiac disease, and liver disease, along with other disorders not traditionally associated with the gut, namely obesity, type 1 and type 2 diabetes, depression, autism, Alzheimer’s, and Parkinson’s disease [5,13,27,42]. It is hypothesized that intestinal barrier function is compromised in intestinal and systemic diseases, which leads to an increase in the level of antigens and bacterial metabolites in human organisms and results in infectious and systemic inflammatory reactions that exacerbate the pathophysiological processes underlying diseases. Disturbances in the intestinal barrier can be associated with both structural damage to the mucosal area and changes in the regulation of structural components of junctional complexes [9,43].

However, it is noteworthy that it has not yet been established whether the loss of barrier integrity is a cause or a consequence of diseases. On the other hand, recent findings show that therapeutic intervention to improve the integrity of the intestinal barrier can reduce the symptoms of several diseases. Therefore, the development of new therapeutic tools for diagnosing mucosal barrier function may hold promise for treating and preventing illnesses [9,11,44,45].

In addition, GI symptoms such as abdominal pain, diarrhea, and flatulence are common symptoms in the general population. However, the source of these symptoms may be IBDs such as Crohn’s disease and ulcerative colitis, as well as collagenous colitis or IBS. Due to the varied and non-specific nature of the general population’s GI symptoms, it may be challenging to differentiate an organic disease from functional disorders in symptomatic patients, and delineating the two may be of crucial importance for an accurate diagnosis. Therefore, to be able to distinguish organic from functional bowel diseases, avoid delays in diagnosis, and reduce unnecessary invasive procedures and costly tests, it is necessary to develop non-invasive diagnostic tools such as biomarkers that are both specific for and sensitive enough to detect disease [46,47,48,49].

To date, several biomarkers in blood, stool, and urine have been developed to provide scientists with potential insight into the gut environment and diagnose gut permeability and epithelial integrity. An understanding of the importance of these biomarkers in healthy and disease states can help with characterizing the pathogenesis of the disease, reaching a precise diagnosis, developing a targeted therapy, and monitoring treatment response. However, the practical use and limitations of biomarkers are currently under extensive discussion [9,10,13,28].

Our study tried to determine whether the levels of calprotectin, zonulin, and sIgA may depend on other additional factors, often not included in the diagnostic analysis. These include gender, age, BMI, the composition of gut microorganisms, probiotic consumption, and GI symptoms such as constipation, diarrhea, bloating, and functional disorders of the GI tract.

Calprotectin is a widely employed biomarker for fecal samples used to detect enteritis and track mucosal barrier function. The presence of calprotectin in the stool appears to be directly related to neutrophil migration into the intestinal lumen due to infection or inflammation. This protein seems to have a regulatory function in inflammatory processes and shows antibacterial and antiproliferative activity [29,48,50,51]. Furthermore, calprotectin levels correlate well with enteritis, and calprotectin is used as a biomarker in GI disorders, as well as for monitoring disease activity and detecting disease recurrences. Numerous studies have shown that calprotectin is a sensitive marker of inflammation in the GI tract and may increase in cases of IBD, Crohn’s disease, and ulcerative colitis. However, the clinical utility of calprotectin in other GI disorders remains unclear [47,50,51,52].

Our study showed that the median CAL concentration in participants was 30 µg/g, while a level below 80 μg/g suggests a negative test result in non-inflammatory diagnostics. However, it should be noted that the standards quoted by test manufacturers often differ, and the cut-off point for a valid value varies between 50 μg/g and 80 μg/g. Conversely, von Roon et al. demonstrated that a cut-off of 100 µg/g may have higher diagnostic efficiency and precision than 50 µg/g [50,53,54,55]. Contrary to other studies, except that of Park et al. [56], we did not observe any correlations between gender, BMI, and calprotectin concentration [54,56,57,58,59].

Additionally, we did not demonstrate a greater concentration of this biomarker in the group with GI symptoms and functional disorders. It should be noted that the data on calprotectin in patients with abdominal discomfort are often inconsistent and heterogeneous, indicating either a higher or an average concentration of this marker in the stool [60,61,62,63]. At the same time, our analysis showed that calprotectin levels could be associated with age. In the group aged >55 years, we observed increased concentrations, which agrees with the observations of Park et al. and Joshi et al. [56,64]. Furthermore, this may support the theory that calprotectin concentrations significantly correlate with age. This is observed not only in adults but in children as well. A wide range of research indicates that the physiological levels of calprotectin in healthy children are higher than in healthy adults [65,66,67,68]. Our findings in adults and other pediatric studies suggest that a separate reference range would likely be required to evaluate calprotectin in children and older adults, but further studies are needed to confirm the proposed values in clinical practice.

Zonulin (haptoglobin 2 precursor) is a newly discovered human analog of zonula occludens protein, an endotoxin secreted by the *Vibrio cholerae*. It is synthesized in the liver and epithelial cells and can be isolated from multiprotein membrane complexes. ZON acts as a modulator that exhibits intercellular tight-junction regulatory activity, binding to a specific receptor on the surface of intestinal epithelia [16,17,69,70]. Excessive production of zonulin triggers a cascade of biochemical events that induce the breakdown of tight junctions, leading to increased permeability of the intestinal epithelium and subsequent immune responses. Therefore, it is presumed that zonulin plays a crucial role in maintaining intestinal mucosal homeostasis by regulating the tight junctions and modulating the transport between the intestinal lumen and the host’s internal environment of molecules weighing at least 3.5 kDa [16,17,24]. It is worth noting that several potential stimuli could lead to an increase in ZON levels However, studies based on cell cultures have shown that exposure to Gram-negative bacteria and gliadin, the main fraction of wheat gluten, can be strong triggers [25,26,71,72,73]. According to recent clinical studies, elevated zonulin levels contribute to the development of various chronic inflammatory diseases, including autoimmune and infectious responses, together with metabolic and cancerous states [16,17,24,74,75,76].

Our results showed that the median zonulin level in participants’ feces was 65 ng/mL. According to the manufacturer’s protocol, the median concentration of 61 ng/mL (±46 ng/mL) is the cut-off for classifying people as being without increased intestinal barrier permeability. It should be noted, however, that according to Massier et al. and Scheffler et al., commercially available ELISA kits do not measure zonulin levels but concentrations of properdin, a protein belonging to the mannose-associated serine protease family. Thus, the tests currently used in research and commercial laboratories do not assess intestinal permeability. Rigorous assays such as dual-sugar assays and the use of immunohistochemistry and zonula occludens proteins expression profiles are needed to measure intestinal permeability, and these assays should be the gold standard for assessing intestinal permeability in vivo [77,78]. We found no correlations of gender, BMI, or age with zonulin levels. Furthermore, we did not detect an altered concentration of this biomarker in participants reporting gastrointestinal symptoms and functional disorders. These results are consistent with other authors, who found no significant relationships between similar factors and ZON concentrations [79,80]. However, in the case of zonulin, it is crucial to select the appropriate diagnostic test. A comparison of similar factors based on circulating ZON shows more positive results than a fecal test. A significant correlation with zonulin serum levels was observed in the cases of BMI, age, and gender [80,81,82]. On the other hand, previous investigations examining the relationship between functional gastric disorders and ZON concentrations have shown both positive [83,84] and negative results [85,86,87], regardless of the type of test used.

Another biomarker, sIgA, is used as a marker of mucosal immunity. Due to properties such as resistance to enzymatic degradation and the ability to survive the harsh conditions of the gastrointestinal tract, as well as its dimeric form enabling cross-linking and entrapment of bacteria, the secretory form of IgA plays a crucial role in mucosal immunity and protects against potentially pathogenic microorganisms. sIgA is produced in response to antigens derived from microbes and food, and it protects mucosal surfaces by binding to viruses and bacteria to prevent or inhibit their adherence to epithelial cells in a process known as immune exclusion [6,88,89,90]. Moreover, sIgA is a crucial component of selecting and maintaining the intestinal microbiota. Approximately 25–75% of sIgA binds to the commensal microorganisms, thus inhibiting their penetration through the intestinal epithelium. It was recently discovered that interactions between sIgA and commensal microbes are bidirectional, and beneficial bacteria may modulate sIgA distribution and regulate adaptive immunity by providing a low level of immune stimulation [10,89,91,92,93]. This is consistent with our observations about the complex interactions of microorganisms, which can positively or negatively influence each other or the levels of the biomarkers themselves.

As far as our analysis is concerned, we showed that the median concentration in feces was 1439 µg/mL and was within the normal range (510 µg/mL and 2040 µg/mL). Furthermore, we saw no association between BMI, gender, the incidence of functional gastrointestinal disorders, and sIgA levels. However, as with calprotectin, we have seen a relationship between age and sIgA concentration, and in the group >55 years, we observed lower sIgA concentrations.

We hypothesize that altered IgA levels might be related to the microbiome changes that occur with advancing age. Growing evidence points to an age-related decline in the diversity of the microbiota, which in the elderly can reduce the effectiveness of the immune system and increase the occurrence of low-grade inflammation. Immunological changes related to aging are associated with a decrease in B- and T-lymphocyte-mediated response and higher concentrations of C-reactive protein and circulating inflammatory cytokines [94,95,96,97,98]. Interestingly, our analysis showed a strong negative correlation between the sIgA value and calprotectin. In connection with the above, it should be considered whether low levels of sIgA in the elderly should be treated as a pathological or physiological condition related to age. On the other hand, it is necessary to consider whether diseases that appear later in life are a cause or a consequence of disturbances in the community of the microbiota and the intestinal barrier.

The reduction of low-grade inflammation may be a way to prevent or slow age-related changes, and probiotic supplementation has been proposed to achieve this. Numerous studies have demonstrated that consuming probiotics can effectively treat age-related dysbiosis, maintaining a healthy gut microbiota and immune homeostasis during aging [96,99,100,101]. According to the latest research, probiotics can stimulate the innate and acquired immune response by inducing secretory and systemic IgA secretion, promoting phagocytosis, modifying T lymphocyte responses, and maintaining Th1 and Th2 responses [101,102,103,104].

We also observed a relationship between sIgA level and probiotic supplementation. However, an enhanced sIgA value was demonstrated in the group not taking probiotics (1917 µg/mL vs. 990 µg/mL for the non-supplemented group). Moreover, 39% of participants in the non-probiotic group had sIgA values above the normal range, while only 15% did in the probiotic group. We speculate that higher levels of sIgA may be associated with an unbalanced, poor commensal bacteria gut microbiota and may indicate inflammation and infection of the digestive tract. Conversely, a lower concentration of sIgA in the non-probiotic consumers may indicate homeostasis between the gut microbes and the immune system. However, we would like to emphasize that this was not a controlled study, and patients independently supplemented various types of probiotics.

The results of numerous studies suggest that balance and diversity within the bacterial population are necessary for maintaining the correct functioning of the GI tract and the host immune system. Enteric microbes play a crucial role in the training and development of the host immune system components and immune responses. They are also involved in many host physiological processes, including shaping the integrity and immunity of the intestinal barrier [43,105,106,107]. There is increasing evidence that a well-balanced microbiota partakes in epithelial barrier function through the production of short-chain fatty acids (SCFA) and the recognition of microorganisms by pattern recognition receptors in the mucosa [10]. In light of this, we decided to investigate whether certain species of intestinal bacteria predispose changes in the levels of biomarkers related to barrier permeability/inflammation/immunity. Since our analysis was based on a small number of bacterial species, we could observe complex relationships between them and intestinal markers. However, due to the limitations and specificity of the method used, we did not assess species and strains belonging to the genus.

Firstly, we found a strong positive correlation between calprotectin levels and *Bifidobacterium* and *Lactobacillus* abundance. However, in this case, we hypothesize that the increased levels of *Bifidobacterium* and *Lactobacillus* may be a compensatory mechanism in the inflammatory process that is already underway. Recently published studies have shown that these groups of bacteria have strong anti-inflammatory properties. Some strains of *Bifidobacterium* can modulate and inhibit the host’s immune response through dendritic cell interactions. They influence the increased production of the anti-inflammatory interleukin 10 and reduce the levels of inflammatory cytokines such as tumor necrosis factor-alpha and interleukin 6 [108,109,110]. In addition, *Bifidobacterium* and *Lactobacillus* produce SCFAs that can bind to specific receptors on intestinal epithelial cells. In this way, the NF-κB pathway is inhibited, together with Tregs and the production of proinflammatory cytokines, which prevents inflammation and induces an anti-inflammatory effect [111].

Additionally, we also showed a strong negative correlation between the value of zonulin and *Lactobacillus* genus. Other studies support our results, showing an association between certain *Lactobacillus* strains and a marker of barrier permeability [76,112,113,114]. It is assumed that commensal and probiotic strains could enhance the intestinal epithelial structural barrier by regulating the expression of tight junctions and prevent or reverse membrane barrier disruption caused by the pathogens [115,116]. In addition, *Lactobacillus* protects the epithelium against colonization by pathogens, inhibits the adhesion of pathogens to the intestinal mucosa, and prevents inflammatory processes. Although the mechanisms by which probiotics and commensal bacteria can enhance gut barrier function are not precise, several studies have shown that they can inhibit epithelial barrier disruption through the mitogen-activated protein kinase (MAPK) signaling pathway or by increased gene expression contributing to encoding binding proteins, such as E-cadherin and β-catenin [110,116].

Through further analysis, we obtained a strong negative correlation between *Citrobacter* spp. and sIgA. *Citrobacter* spp. belongs to the *Proteobacteria* type, which has a strong influence on the maturation of the intestinal microbiota community and the development of mucosal and systemic immunity [117,118]. *Proteobacteria* are the dominant group in the neonatal period, taking part in the development of immune tolerance and thus significantly influencing the functioning of the microbiome in adulthood [88,117,118,119]. On the other hand, a disturbing process of “maturation” of the intestinal ecosystem in early childhood may cause the same species of bacteria to have a different effect on the intestinal immune system, leading to inflammation and a weakening of intestinal immunity [119,120]. Our results also showed a positive correlation between sIgA and *Enterococcus* genus. This genus includes various species and strains that can positively and negatively affect the human body. *Enterococcus* species, as commensals, can colonize the digestive system and participate in the development of the immune system. Like probiotic strains, they can stimulate sIgA secretion in a non-inflammatory reaction [121,122]. However, excessive growth of these bacteria resulting from disturbed intestinal homeostasis may lead to disruptions of intestinal barrier function, spontaneous translocation of bacteria, long-term immune response, and, consequently, the development of pathological disease states [123,124].

However, in our opinion, the results obtained in our study should be taken with caution due to several limitations. The study’s primary limitation is the small group of respondents and a limited number of species analyzed. Moreover, many patients were excluded from further analysis because they did not meet the inclusion criteria. Additionally, it was not a controlled study, and some patients did not complete all the questions and the required diagnostic tests. The number of patients presenting some of the required variables was also insufficient. Therefore, it is not easy to draw definitive conclusions about these parameters. Moreover, the questionnaire did not include lifestyle-related risk factors such as nutrition, smoking, and alcohol consumption. At the same time, some recent studies have shown that these factors may contribute to higher levels of biomarkers. Despite numerous limitations, we believe that the presented results will be of interest to the scientific community, due to the inclusion of more biomarkers than in other studies, the assessment of correlations between them, and the consideration of many factors that may affect the level of intestinal biomarkers and are often not included in other studies (e.g., selected intestinal microorganisms, consumption of probiotics).

## 5. Conclusions

In conclusion, this study shows that determining the correct cut-off value for biomarkers can be critical for different age groups. Currently, reference values are used regardless of the age of the patients. Therefore, for some biomarkers, especially calprotectin and sIgA, scrupulous standardization within age groups should be performed to improve the performance of diagnostic tests. No correlation was observed between BMI, gender, functional disorders of the gastrointestinal tract, and the concentration of analyzed biomarkers. Our findings also support a possible causal link between qualitative changes in gut microorganisms and biomarker levels. Nevertheless, the link between microbial interactions and biomarkers requires further research to determine the exact associations between these two factors.

## Figures and Tables

**Table 1:** Reference ranges for biomarkers, interpretation, and detection limits.

Biomarker	Ranges	Interpretation	Limit of Quantification
Calprotectin	<80 µg/g	Normal	Lower: ≤28.2 μg/g
	80–160 µg/g	Borderline/gray zone	
	>160 µg/g	Elevated	Upper: ≥1002 μg/g
Zonulin	≤105 ng/mL	Normal	Lower: ≤0.241 ng/mL
	>105 ng/mL	Elevated	Upper: ≥170 ng/mL
sIgA	<510 µg/mL	Subnormal	Lower: ≤310 µg/mL
	510–2040 µg/mL	Normal	
	>2040 µg/mL	Elevated	Upper: N/A ^a^

**Legend:**^a^—the upper limit was not available (N/A); samples above the reference standard curve values were diluted with wash buffer and measured again.

**Table 2:** Analyzed microorganisms and the reference values according to the Institute of Microecology (Herborn, Germany).

Genera	Reference Values
*Escherichia coli*	≥10^6^
*Escherichia coli* (NLF)	<2 × 10^4^
*Proteus* spp.	<2 × 10^4^
*Providencia* spp.	<2 × 10^4^
*Morganella* spp.	<2 × 10^4^
*Pseudomonas* spp.	<2 × 10^4^
*Klebsiella* spp.	<2 × 10^4^
*Enterobacter* spp.	<2 × 10^4^
*Citrobacter* spp.	<2 × 10^4^
*Serratia* spp.	<2 × 10^4^
*Hafnia alvei*	<2 × 10^4^
*Enterococcus* spp.	≥10^6^
*Bifidobacterium* spp.	≥10^9^
*Bacteroides* spp.	≥10^9^
*Lactobacillus* spp.	≥10^5^
H_2_O_2_ *Lactobacillus*	≥10^5^
*Clostridium* spp.	≤10^5^
*Faecalibacterium prausnitzii*	≥10^9^
*Akkermansia muciniphila*	≥10^8^
Total bacteria count (TBC)	≥10^11^
*Candida* spp.	<10^3^

Lower detection limit = 10^3^, upper detection limit = 10^12.^

## Data Availability

The data that support the findings of this study are available on reasonable request from the corresponding author. The data are not publicly available due to privacy or ethical restrictions.

## Supplementary Materials

The following supporting information can be downloaded at https://www.mdpi.com/article/10.3390/biom12121781/s1. Table S1: Characteristics of the participants. Table S2: Biomarker level characteristics in participants’ stool. Table S3: Summary of analyzed microbiota characteristics regarding probiotic supplementation. Table S4: Significant correlations between analyzed biomarkers and selected microorganisms in all studied cases. Table S5: Significant correlations between analyzed biomarkers and selected microorganism groups distinguished according to probiotics usage.
