# Supplementary material for: Impact of Biometric Patient Data, Probiotic Supplementation, and Selected Gut Microorganisms on Calprotectin, Zonulin, and sIgA Concentrations in the Stool of Adults Aged 18–74 Years
Source: Biomolecules. 2022 Nov 29;12(12):1781. doi: 10.3390/biom12121781 (PMC9775524; doi:10.3390/biom12121781)
Supplement: Supplementary file 1 [file biomolecules-12-01781-s001.zip › biomolecules-2040642-supplementary.pdf]

Impact of Biometric Patient Data, Probiotic Supplementation, and Selected Gut Microorganisms on Calprotectin, Zonulin, and sIgA Concentrations in the Stool of Adults Aged
18–74 Years

Magdalena Jendraszak [1,*], Mirosława Gałęcka [2], Małgorzata Kotwicka [1], Andreas Schwiertz [3], Aleksandra Regdos [2], Michalina Pazgrat-Patan [2] and Mirosław Andrusiewicz [1,*]

[1] Chair and Department of Cell Biology, Poznan University of Medical Sciences, Rokietnicka 5D, 60-806 Poznań, Poland
[2] Institute of Microecology, Sielska 6, 60-129 Poznań, Poland
[3] Institute of Microecology, Auf den Lüppen 8, 35745 Herborn, Germany
* Correspondence: jendraszak@ump.edu.pl (M.J.); andrus@ump.edu.pl (M.A.)

Supplementary tables

Table S1. Characteristics of the participants.

	Grouping variable	N	M	SD	Me	Q1	Q3	*p*-value
Age	-	160	40	12.7	39	31	47	-
Body mass [kg]		138	69	15.9	66	58	78	
BMI		138	23.41	4.65	22.53	20.38	25.86	
Sex								
Age	female	111	41	13.1	39	31	48	.3300[a]
	male	49	38	11.7	38	31	44	
Body mass [kg]	female	97	65	15.4	63	54	78	**<.0001**[a]
	male	41	79	12.8	75	70	90	
BMI	female	97	23.09	4.91	21.94	19.82	24.80	**.0413**[a]
	male	41	24.17	3.91	23.89	21.22	26.86	
Probiotics usage								
Age	Non-supplementation	62	39	11.3	38	31	44	.6959[b]
	Supplementation	45	40	13.3	37	31	47	
Body mass [kg]	Non-supplementation	59	71	17	70	58	86	.1548[b]
	Supplementation	44	66	13	65	59	74	
BMI	Non-supplementation	59	24.18	5.60	23.53	20.31	26.88	.2627[a]
	Supplementation	44	22.29	3.37	22.36	20.06	24.55	
	Present	86	23.10	4.149	22.44	20.38	25.61	

Legend: N – number of participants; M – Mean; SD – Standard deviation; Me – Median, Q1, Q3 – Lower and Upper Quartile; [a] – Mann-Whitney U test; [b] – t-Student test; $p<0.05$ are indicated in bold

Table S2. Biomarkers level characteristics in participants' stool.

Marker	Grouping variable	N	M	SD	Me	Q1	Q3	*p*-value
Calprotectin ug/g	-	74	85	180.1	30	30	42	-
sIgA ug/ml		51	1702	1462	1399	549	2241	
Zonulin ng/ml		80	85.53	63.27	71.70	48.19	102.95	
Probiotics usage								
Calprotectin μg/g	Non-supplementation	29	100	232.6	30	30	35	<.9999[a]
	Supplementation	21	69	91.5	30	30	36	
sIgA μg/ml	Non-supplementation	18	1917	1567.5	1868	914	2290	**.0464[b]**
	Supplementation	13	990	690.2	686	494	1653	
Zonulin ng/ml	Non-supplementation	31	96.34	56.341	83.26	57.70	119.50	.1819[a]
	Supplementation	25	85.61	76.19	69.98	48.58	102.00	
Gastrointestinal disorders presence								
Calprotectin μg/g	Absent	18	109	197.4	32	30	107	.0712[a]
	Present	49	65	148.1	30	30	35	
sIgA μg/ml	Absent	20	1847	1486.3	1419	671	2274	.3762[a]
	Present	29	1567	1439.9	1305	442	2121	
Zonulin ng/ml	Absent	35	81.05	52.853	68.49	45.39	86.90	.6379[a]
	Present	42	89.21	72.718	73.50	48.70	104.10	

Legend: N – number of participants; M – Mean; SD – Standard deviation; Me – Median, Q1, Q3 – Lower and Upper Quartile; [a] – Mann-Whitney U test; [b] – t-Student test; $p<0.05$ are indicated in bold

Table S3. Summary of analyzed microbiota characteristics regarding probiotic supplementation.

Microbiota	Probiotics												*p*-value[a]
	**Non-supplementation**						**Supplementation**						
	N	M	SD	Me	Q1	Q3	N	M	SD	Me	Q1	Q3	
Escherichia coli	61	2.16E7	4.03E7	4.00E6	8.00E5	3.00E7	45	2.14E7	4.46E7	4.00E6	1.00E6	1.00E7	.8746
Escherichia coli (NLF)	62	1.15E5	4.74E5	2.00E3	2.00E3	2.00E3	45	1.43E6	4.38E6	2.00E3	2.00E3	2.00E3	**.0291**
Pseudomonas spp.	62	5.19E3	2.51E4	2.00E3	2.00E3	2.00E3	44	2.01E4	8.66E4	2.00E3	2.00E3	2.00E3	.4009
Klebsiella spp.	61	3.41E5	1.47E6	2.00E3	2.00E3	2.00E3	45	2.73E5	1.32E6	2.00E3	2.00E3	2.00E3	.2627
Enterobacter spp.	62	6.33E5	1.76E6	2.00E3	2.00E3	2.00E3	45	1.08E5	4.22E5	2.00E3	2.00E3	2.00E3	.9135
Citrobacter spp.	62	1.70E5	1.27E6	2.00E3	2.00E3	2.00E3	45	1.56E6	6.64E6	2.00E3	2.00E3	2.00E3	.1650
Enterococcus spp.	62	3.36E7	1.18E8	2.00E3	2.00E3	8.00E5	45	4.18E7	1.15E8	2.00E3	2.00E3	6.00E6	.7951
Bifidobacterium spp.	62	1.04E9	9.37E8	8.00E8	4.00E8	1.00E9	45	8.77E8	9.67E8	6.00E8	2.00E8	1.00E9	.6872
Bacteroides spp.	62	2.39E9	2.02E9	2.00E9	1.00E9	3.00E9	45	2.24E9	1.64E9	2.00E9	1.00E9	3.00E9	.2819
Lactobacillus spp.	62	3.86E5	9.69E5	3.00E4	2.00E3	3.00E5	45	8.93E5	2.13E6	6.00E4	2.00E3	7.00E5	.7050
H_2O_2 *Lactobacillus*	62	9.70E4	3.04E5	2.00E3	2.00E3	4.00E4	44	2.09E5	5.66E5	2.00E3	2.00E3	7.50E4	.9246
Clostridium spp.	61	1.77E4	4.91E4	2.00E3	2.00E3	2.00E3	45	7.36E4	2.26E5	2.00E3	2.00E3	2.00E3	.1315
Faecalibacterium prausnitzii	62	4.57E9	3.69E9	3.00E9	2.00E9	7.00E9	45	5.24E9	4.45E9	4.00E9	2.00E9	8.00E9	<.9999
Akkermansia muciniphila	62	8.06E7	1.75E8	6.00E6	8.00E3	8.00E7	45	9.40E7	2.01E8	7.00E6	1.00E2	7.00E7	.3422
TBC	62	1.38E10	1.42E10	1.00E10	5.00E9	2.00E10	45	1.03E10	1.06E10	7.00E9	4.00E9	1.00E10	.7926
Candida spp.	62	1.74E3	5.43E3	1.00E2	1.00E2	5.00E2	44	4.71E3	1.52E4	1.00E2	1.00E2	1.00E3	.7037

Legend: N – number of participants; M – Mean; SD – Standard deviation; Me – Median, Q1, Q3 – Lower and Upper Quartile; [a] – Mann-Whitney U test; $p<0.05$ are indicated in bold

Table S4. Significant correlations between analyzed biomarkers and selected microorganisms in all studied cases.

From	To	R	*p*-value
Calprotectin µg/g	H_2O_2 *Lactobacillus*	0.25	**.0343**
Calprotectin µg/g	*Bifidobacterium* spp.	0.25	**.0030**
sIgA µg/ml	*Enterococcus* spp.	0.28	**.0447**
sIgA µg/ml	*Citrobacter* spp.	-0.39	**.0042**
sIgA µg/ml	Calprotectin µg/g	-0.60	**.0085**
Zonulin ng/ml	*Lactobacillus* spp.	-0.27	**.0145**
Bacteroides spp.	TBC	0.58	**<0001**
Bacteroides spp.	*Pseudomonas* spp.	0.19	**.0158**
Bacteroides spp.	*Bifidobacterium* spp.	0.36	**<.0001**
Bifidobacterium spp.	TBC	0.50	**<.0001**
Candida spp.	*Faecalibacterium prausnitzii*	0.18	**.0232**
Candida spp.	*Lactobacillus* spp.	0.19	**.0197**
Candida spp.	H_2O_2 *Lactobacillus*	0.21	**.0067**
Citrobacter spp.	*Escherichia coli* (NLF)	0.16	**.0465**
Enterobacter spp.	*Escherichia coli*	0.16	**.0391**
Enterococcus spp.	*Citrobacter* spp.	0.16	**.0471**
Enterococcus spp.	*Escherichia coli*	0.16	**.0454**
Escherichia coli	*Escherichia coli* (NLF)	-0.17	**.0319**
Faecalibacterium prausnitzii	*Bifidobacterium* spp.	0.16	**.0378**
Faecalibacterium prausnitzii	TBC	0.30	**<.0001**
H_2O_2 *Lactobacillus*	*Escherichia coli* (NLF)	0.22	**.0052**
H_2O_2 *Lactobacillus*	*Bifidobacterium* spp.	0.24	**.0021**
H_2O_2 *Lactobacillus*	*Lactobacillus* spp.	0.61	**<.0001**
Lactobacillus spp.	*Escherichia coli*	0.18	**.0249**
Lactobacillus spp.	*Bifidobacterium* spp.	0.22	**.0048**
Lactobacillus spp.	*Escherichia coli* (NLF)	0.23	**.0031**
Lactobacillus spp.	*Enterococcus* spp.	0.31	**<.0001**
Lactobacillus spp.	TBC	0.18	**.0204**

Legend: R – Spearman's rank coefficient of correlation; TBC – total bacteria count; NLF – non-lactose fermenting; $p<0.05$ are indicated in bold

R values ranges:	-1 | -0.8	-0.79 | -0.6	-0.59 | -0.4	-0.39 | -0.2	-0.19 | 0 | 0.19	0.2 | 0.39	0.4 | 0.59	0.6 | 0.79	0.8 | 1
Proportionality	Negative/inversely proportional				No correlation	Positive/directly proportional			
Strength Interpretation	very strong	strong	moderate	weak	very weak	weak	moderate	strong	very strong

Table S5. Significant correlations between analyzed biomarkers and selected microorganisms groups distinguished probiotics usage accordingly.

**Probiotics non-supplementation**				**Probiotics supplementation**			
From	To	R	*p*-value	From	To	R	*p*-value
Calprotectin μg/g	*Klebsiella* spp.	0.44	**.0203**	Calprotectin μg/g	*Escherichia coli*	0.43	**.0496**
Zonulin ng/ml	*Lactobacillus* spp.	-0.43	**.0164**	Calprotectin μg/g	*Lactobacillus* spp.	0.47	**.0304**
Zonulin ng/ml	*Clostridium* spp.	0.36	**.0468**	Calprotectin μg/g	H_2O_2 *Lactobacillus*	0.64	**.0024**
Bacteroides spp.	*Bifidobacterium* spp.	0.51	**<.0001**	Zonulin ng/ml	*Escherichia coli* (NLF)	0.40	**.0468**
Bacteroides spp.	TBC	0.67	**<.0001**	*Akkermansia muciniphila*	*Enterobacter* spp.	0.37	**.0114**
Candida spp.	*Bacteroides* spp.	0.33	**.0084**	*Bacteroides* spp.	*Bifidobacterium* spp.	0.37	**.0127**
Candida spp.	TBC	0.28	**.0294**	*Bacteroides* spp.	TBC	0.62	**<.0001**
Bifidobacterium spp.	TBC	0.57	**<.0001**	*Bifidobacterium* spp.	TBC	0.52	**.0003**
Faecalibacterium prausnitzii	*Candida* spp.	0.34	**.0066**	*Candida* spp.	*Lactobacillus* spp.	0.41	**.0052**
Faecalibacterium prausnitzii	TBC	0.39	**.0019**	*Candida* spp.	H_2O_2 *Lactobacillus*	0.45	**.0027**
H_2O_2 *Lactobacillus*	*Lactobacillus* spp.	0.62	**<.0001**	*Escherichia coli* (NLF)	*Escherichia coli*	-0.40	**.0068**
Lactobacillus spp.	*Enterococcus* spp.	0.29	**.0238**	*Faecalibacterium prausnitzii*	*Bifidobacterium* spp.	0.34	**.0234**
				Faecalibacterium prausnitzii	TBC	0.43	**.0031**
				H_2O_2 *Lactobacillus*	*Lactobacillus* spp.	0.62	**<.0001**
				Lactobacillus spp.	*Enterococcus* spp.	0.33	**.0262**
				Lactobacillus spp.	*Bifidobacterium* spp.	0.34	**.0242**
				Lactobacillus spp.	TBC	0.36	**.0155**

Legend: R – Spearman's rank coefficient of correlation; TBC – total bacteria count; NLF – non-lactose fermenting; *p*<0.05 are indicated in bold

R values ranges:	-1	-0.8	-0.79	-0.6	-0.59	-0.4	-0.39	-0.2	-0.19	0	0.19	0.2	0.39	0.4	0.59	0.6	0.79	0.8	1
Proportionality	Negative/inversely proportional									No correlation	Positive/directly proportional								
Strength Interpretation	very strong		strong		moderate		weak		very weak			weak		moderate		strong		very strong	
